# The Function of Chitinases CmCH1 and CmCH10 in the Interaction of *Coniothyrium minitans* and *Sclerotinia sclerotiorum*

**DOI:** 10.3390/ijms26178706

**Published:** 2025-09-06

**Authors:** Haixuan Wang, Huizhang Zhao, Zihang Zhu, Yang Lin, Jiatao Xie, Jiasen Cheng, Daohong Jiang, Yanping Fu

**Affiliations:** 1 The Provincial Key Lab of Plant Pathology of Hubei Province, College of Plant Science and Technology, Huazhong Agricultural University, Wuhan 430070, China; wanghx117@163.com (H.W.); zhuzihang@webmail.hzau.edu.cn (Z.Z.); yanglin@mail.hzau.edu.cn (Y.L.); jiataoxie@mail.hzau.edu.cn (J.X.); daohongjiang@mail.hzau.edu.cn (D.J.); 2 Industrial Crops Institute, Hubei Academy of Agricultural Sciences, Wuhan 430064, China; huizhangfungi@hbaas.ac.cn; 3 National Key Laboratory of Agricultural Microbiology, Huazhong Agricultural University, Wuhan 430070, China

**Keywords:** *Coniothyrium minitans*, *Sclerotinia sclerotiorum*, chitinase, gene function

## Abstract

*Sclerotinia sclerotiorum*, a devastating phytopathogenic fungus with global distribution, exhibits a broad host range encompassing over 700 plant species. Sclerotinia stem rot caused by this pathogen poses a significant threat to sustainable oilseed rape production. *Coniothyrium minitans*, a mycoparasite of *S. sclerotiorum*, is a promising biological control agent against this devastating disease. *C. minitans*-based formulations have been commercially developed for field application. A transcriptomic analysis revealed significant upregulation of the chitinase-encoding gene *CmCH1* in *C. minitans* during interaction with *S. sclerotiorum.* Knockout of either *CmCH1* or another chitinase-encoding gene *CmCH10* in *C. minitans* did not markedly affect the mycelial growth, development, and parasitism of *S. sclerotiorum*. However, knockout *CmCH1* and *CmCH10* simultaneously resulted in reduced growth rate, impaired protoplast release, enhanced cell wall integrity, and diminished mycoparasitic capability. These results indicate that *CmCH1* and *CmCH10* collectively influence remodeling of the cell wall in *C. minitans* and its mycoparasitic activity.

## 1. Introduction

*Sclerotinia sclerotiorum*, a globally distributed plant pathogen, infects over 700 plant species and causes severe annual yield losses worldwide [1,2,3]. Disease management remains particularly challenging due to its broad host range, remarkable field persistence, and the lack of resistant cultivars [1,4]. The mycoparasitic fungus *Coniothyrium minitans* has been successfully developed as a biocontrol agent against *S. sclerotiorum*, demonstrating consistent field efficacy in disease suppression [5,6,7,8].

Current research has revealed that *C. minitans* employs a sophisticated multi-component strategy to control *S. sclerotiorum* through coordinated genetic regulation and biochemical mechanisms. The parasitic process is governed by an intricate network of pathways regulating morphological development, conidiation, antimicrobial production, stress resistance, and hydrolase synthesis. First, *C. minitans* produces bioactive secondary metabolites to effectively inhibit competing microorganisms. The broth contains at least two distinct classes of antifungal agents with differential physicochemical properties, namely a heat-stable fraction targeting *S. sclerotiorum* and a thermolabile proteinaceous component active against *Colletotrichum higginsianum* [9,10]. Genetic studies have identified several key regulatory elements. The MAPK signaling pathway genes (*CmSTE7*, *CmBCK1*, *CmSlt2*, and *CmNox1*) modulate conidiation and parasitic efficiency [11,12]. Furthermore, *C. minitans* secretes chitinases and β-1,3-glucanases to effectively degrade the various cellular structures of *S. sclerotiorum* [13]. These cell wall-degrading enzymes (CWDEs), encoded by *CmCH1* and *Cmg1*, play a crucial role in host penetration [14]. Their expression is markedly suppressed in parasitic-defective mutants but is strongly induced by *S. sclerotiorum*, with *CmCH1* exhibiting particularly significant upregulation, indicating a sophisticated host recognition and response mechanism [12,15,16].

Chitinases represent a class of hydrolytic enzymes that catalyze the breakdown of chitin into N-acetyl-oligosaccharides and glucose, widely distributed in nature [17]. Based on their amino acid sequences and three-dimensional structural characteristics, chitinases are primarily classified into glycoside hydrolase families 18 (GH18), 19 (GH19), and 20 (GH20). Among them, family GH18 has numerous members and is widely present in nature. Although the classification of chitinase is relatively complex, its basic structure is relatively consistent. The structure of chitinase, from the N-terminus to C-terminus, includes a signal peptide, chitin catalytic domain, chitin-binding domain (ChtBD), LysM domain, and a functionally unknown C-terminus. The differences in these domains give chitinase different functions and characteristics [18]. Although the ChtBD domain cannot hydrolyze chitin, it enhances substrate affinity and thereby improves hydrolysis efficiency. Since the 1980s, numerous chitinase-encoding genes have been successfully cloned and characterized from diverse fungal species, including *Trichoderma* spp., *Aspergillus* spp., *Penicillium* spp., *Chaetomium* spp., *Mucor* spp., *Gliocladium roseum*, *Talaromyces flavus*, *Magnaporthe oryzae*, and *C. minitans*. Comparative genomic analyses reveal significant interspecies variation in both the repertoire and copy numbers of chitinase genes. Notably, pathogenic and mycoparasitic fungi typically possess substantially more chitinase genes than yeast species, reflecting their specialized ecological adaptations [19]. The expression of these genes is subject to sophisticated regulatory control: while being negatively regulated by their hydrolysis end-products, chitinase genes can be induced by chitin, chitosan, or their oligomeric derivatives. Conversely, their expression is repressed by glucose and N-acetylglucosamine through catabolite repression mechanisms [20,21,22,23].

Chitinase genes have been established as molecular markers for mycoparasitism due to their significant upregulation in several well-characterized fungal parasitic systems. A classic example is the marked induction of the *ech-42* gene in *T. harzianum* during its parasitism of *Botrytis cinerea* [24]. Similarly, *CmCH1* has been consistently identified as highly expressed during parasitism on *S. sclerotiorum*, establishing it as a marker gene for this particular parasitic interaction [25,26]. Beyond their role in fungal–fungal interactions, chitinases are recognized as potential defense-related genes conferring resistance against fungal pathogens in plants [27]. Experimental evidence demonstrates that the heterologous expression of chitinases can significantly enhance plant pathogen resistance. For instance, the overexpression of *PbChia1* from *Plasmodiophora brassicae* was shown to improve broad-spectrum disease resistance in *Arabidopsis thaliana* [28], while a constitutive expression *CmCH1* of *C. minitans* enhanced soybean resistance to *S. sclerotiorum* [29].

Despite these advances, the functional mechanisms of chitinases in mediating the parasitic activity of *C. minitans* and regulating its own growth and development remain poorly understood. There may be a synergistic effect among chitinases in *C. minitans*, which requires the involvement of multiple genes to collectively influence the strain’s growth, development, and parasitic capability of *C. minitans*. This study focuses on the *C. minitans*–*S. sclerotiorum* interaction system to elucidate the functional characteristics of chitinases, aiming to provide a theoretical foundation for future applications of *C. minitans*-derived chitinases in controlling Sclerotinia stem rot diseases.

## 2. Results

### 2.1. Identification of Chitinase-Encoding Genes in C. minitans

**Figure 1:**
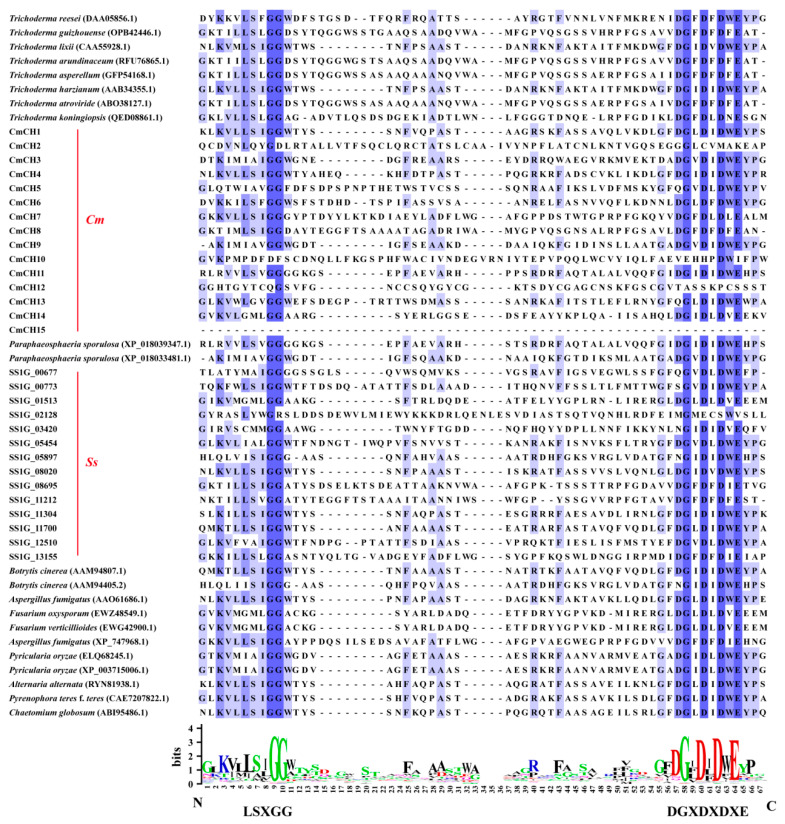
Multiple sequence alignment among chitinases. The amino acid sequence alignment of chitinases was performed by using Jalview software (v 2.11.1.0). The conserved domain of GH18 is expressed in the form of percentage identity, with darker colors indicating more conserved amino acid sites. Conservative amino acid sites are displayed in the form of logos under the sequence, with larger letters indicating more conserved sites. *Cm* means *C. minitans* and *Ss* means *S. sclerotiorum*.

There are fifteen chitinase-encoding genes in the *C. minitans* genome, including the previously characterized *CmCH1*. The other 14 chitinase-encoding genes are systematically designated as *CmCH2* to *CmCH15*. These enzymes exhibited variations in length (108–1561 aa), molecular masses (12.8–163.2 kDa), and isoelectric points (4.32–9.13) (Supplementary Table S2). Phylogenetic analysis revealed that all fifteen chitinases belonged to the GH18 family and shared a distant evolutionary relationship (Figure S1A). Sequence alignment identified “LSXGG” and “DGXDXDXE” as motifs conserved in fungal chitinases, which encompass residues critical for catalytic activity (Figure 1). Experimental validation of the signal peptide-containing chitinases demonstrated secretory activity in nine of them (Figure S1B).

**Figure 2:**
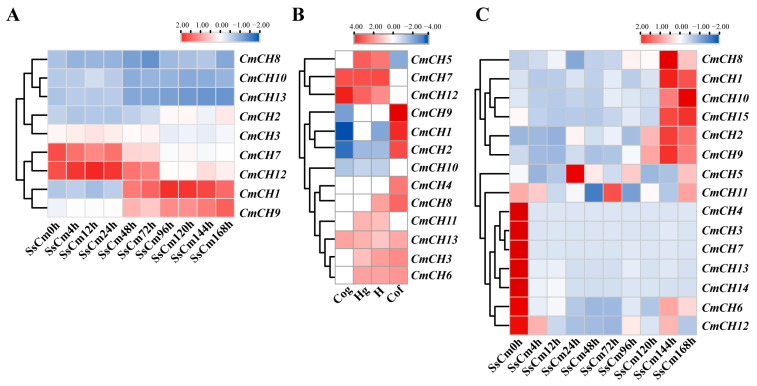
Expression of chitinase genes in *C. minitans*. (**A**) Expression of nine chitinase genes in the transcriptome of *C. minitans* interacting with *S. scleoriorum*. The results were displayed using FPKM by log2 and were subjected to row normalization processing. (**B**) Expression of chitinase genes in the transcriptome of *C. minitans* at different growth stages. Cog: conidial germination (24 hpi), Hg: hyphal growth (36 hpi), H: late hyphal growth (48 hpi), and Cof: conidial formation (72 h). The results were displayed using RPKM by log2 and subjected to row normalization processing. (**C**) Expression of fifteen chitinase genes of *C. minitans* interacting with *S. sclerotiorum* by qRT-PCR. *C. minitans Actin* serves as the internal reference gene. Fold changes in gene expression are shown in color according to the scale. Primer information can be found in Table S1.

Nine chitinase genes were detected and showed different expression patterns during the mycoparasitic interaction, with *CmCH1* showing progressive activation and marked upregulation at the middle and late stages. Additionally, the expression of *CmCH2* and *CmCH9* were upregulated during the late stage of parasitism, whereas *CmCH3*, *CmCH7*, and *CmCH12* were upregulated at the early stages. No significant changes were observed in the expression of *CmCH8*, *CmCH10*, and *CmCH13* (Figure 2A). Based on previous transcriptome sequencing data [29], the expression profiles of chitinase genes in *C. minitans* during the growth stages were also analyzed. The results revealed the expression of thirteen chitinase genes across four developmental phases, including conidial germination (Cog, 24 hpi), hyphal growth (Hg, 36 hpi), late hyphal growth (H, 48 hpi), and conidial formation (Cof, 72 h). During the Cog stage, *CmCH7*, *CmCH12*, and *CmCH13* exhibited enhanced expression levels. At the Hg stage, besides the highly expressed *CmCH5*, *CmCH7*, *CmCH12*, *CmCH3*, *CmCH6*, *CmCH11*, and *CmCH13* were also upregulated. During the H stage, *CmCH8* was additionally upregulated along with the aforementioned seven genes. Notably, during the Cof stage, eight genes (*CmCH1*, *CmCH2*, *CmCH3*, *CmCH4*, *CmCH6*, *CmCH8*, *CmCH9*, and *CmCH13*) exhibited upregulated expression, among which *CmCH1*, *CmCH2*, *CmCH4*, and *CmCH9* displayed stage-specific expression exclusively in this phase (Figure 2B). To further clarify the roles of chitinase genes in the interaction between *C. minitans* and *S. sclerotiorum*, the expression of fifteen chitinase genes in *C. minitans* was detected using qRT-PCR. Although slight discrepancies were observed between qRT-PCR results and transcriptome data, the overall expression trends aligned. Specifically, *CmCH3*, *CmCH4*, *CmCH6*, *CmCH7*, *CmCH12*, *CmCH13*, and *CmCH14* remained unexpressed or minimally expressed, while *CmCH5* and *CmCH11* were undetected in transcriptome analysis but showed detectable expression in qRT-PCR (Figure 2C). In summary, *CmCH1* possesses a functional signal peptide and features a typical chitinase structural domain. Its significant stage-specific expression across various developmental stages suggests a critical role in both the conidiation and parasitism processes of *C. minitans*.

### 2.2. Disruption of CmCH1 Has No Influence on Mycelial Growth and Mycoparasitism of C. minitans

**Figure 3:**
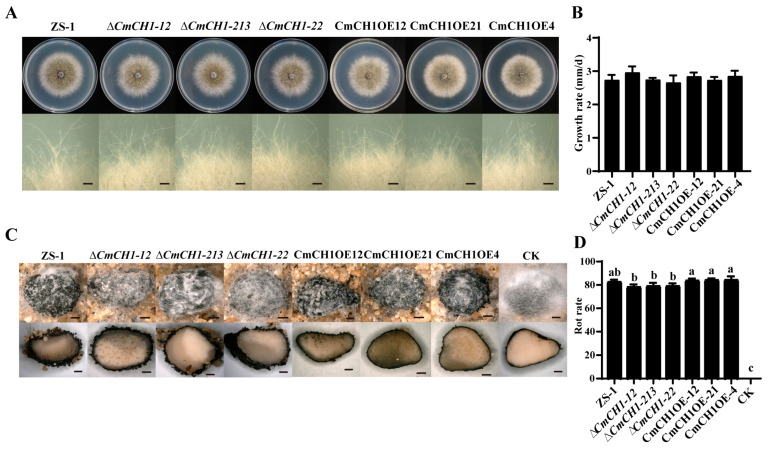
Mycelial growth and parasitic ability of *C. minitans*. (**A**) Colony morphology (20 °C, 10 d) and mycelial tips (20 °C, 5 d) of *C. minitan* on PDA. Bars = 1 mm. (**B**) The growth rate of *C. minitans*. The colony diameter was measured from 2 dpi to 10 dpi. n = 4. (**C**) The surface and crosses of sclerotia parasitized by *C. minitan* (20 °C, 30 d). Sclerotia treated with sterile water served as the control. Bars = 1 mm. (**D**) The rot indices of sclerotia treated with conidial suspension of *C. minitans*. The sclerotia in the CK group showed no decay, resulting in a rot index of 0; n = 4. All values are represented as the average derived from multiple repetitions, with error bars representing ± SD of the average. Different lowercase letters indicate significant differences between strains. One-way ANOVA, *p* < 0.05.

*CmCH1* (*CMZSB_00640*) has a total length of 1384 base pairs (bp), containing one intron and encoding a protein of 444 amino acids. To further investigate the specific role of *CmCH1* in the growth, development, and parasitic processes of *C. minitans*, gene knockout was performed using the homologous recombination split marker strategy, while gene overexpression was achieved through *Agrobacterium*-mediated transformation. Three knockout mutants (*∆CmCH1-12*, *∆CmCH1-213,* and *∆CmCH1-22*) and three overexpression mutants (CmCH1OE12, CmCH1OE21, and CmCH1OE4) were obtained. Compared with the WT strain, three *CmCH1* knockout and three overexpression mutants showed no significant differences in the colony, hyphal tip morphology on PDA (Figure 3A), and growth rate (Figure 3B).

To elucidate the impact of *CmCH1* on the mycoparasitic capability of *C. minitans*, the parasitism capacity of *CmCH1* mutants against the mycelia and sclerotia of *S. sclerotiorum* strain 1980 was evaluated. Strains 1980 and *C. minitans* were co-cultured on PDA for 30 days, and the parasitic capability of *C. minitans* was assessed by colonization on the colony of strain 1980 and the expansion range of pycnidia along the host hypha. The results showed that *CmCH1* knockout and overexpression mutants successfully parasitized the mycelia of strain 1980, forming black pycnidia and abundant conidia (Figure S2A). On the dual-culture plates, the area between the inoculation sites of *C. minitans* and the advancing hyphal front of strain 1980 was evenly divided into four zones (Zones I–IV), and fungal species in each zone were identified (Figure S2B). Hypha isolated and collected from Zones I and II were confirmed to be *C. minitans*, whereas *S. sclerotiorum* was detected in Zones III and IV (Figure S2C). The results showed that the ability of all mutants to parasitize *S. sclerotiorum* on PDA was not affected by the expression of *CmCH1*.

To eliminate the potential interference from nutrients in PDA on the mycoparasitic capability of *C. minitans*, an additional dual-culture experiment was conducted on water agar, where *C. minitans* exhibits minimal autonomous growth. The parasitic capacity of *C. minitans* was evaluated by measuring both the colonization and conidiation on the colony of *S. sclerotiorum*. The results demonstrated that under nutrient-restricted conditions with *S. sclerotiorum* as the exclusive nutrient source, all tested strains, including *CmCH1* knockout mutants, overexpression mutants, and the WT ZS-1 strain, maintained normal growth and conidiation capacity (Figure S2D). No significant differences were observed in the expansion range of pycnidia produced by mutants and ZS-1 on strain 1980 (Figure S2E). Notably, *CmCH1* overexpression mutants produced significantly more conidia compared to both the WT ZS-1 and knockout mutants, while the conidiation of knockout mutants did not differ significantly from those of the ZS-1 (Figure S2F). These results indicate that the overexpression of *CmCH1* significantly enhances the conidiation of *C. minitans* when grown exclusively with *S. sclerotiorum* as the sole nutrient source.

To investigate the impact of *CmCH1* on sclerotial parasitism capability, uniformly sized sclerotia of *S. sclerotiorum* were treated with conidial suspensions (1 × 10^6^ conidia/mL) of *CmCH1* mutants and strain ZS-1, followed by incubation under humid conditions for 30 days. Parasitic activity was evaluated by observing pycnidial formation on sclerotia and calculating the decay index. The results demonstrated that all *C. minitans* strains could normally colonize and form pycnidia on the sclerotia of *S. sclerotiorum* (Figure 3C). The rot indices induced by the three *CmCH1* knockout mutants were 78.48 ± 1.72%, 79.31 ± 2.16%, and 79.25 ± 1.86%, respectively, showing no significant difference from that of ZS-1 (82.87 ± 1.45%). Similarly, the rot indices caused by three overexpression mutants (84.08 ± 1.22%, 84.49 ± 0.97%, and 84.56 ± 2.41%) were not statistically different from that of ZS-1 (Figure 3D). However, *CmCH1* overexpression mutants induced significantly higher sclerotial rot than knockout mutants. Therefore, it was speculated that the disruption of a single chitinase gene may not be able to cause significant changes in *C. minitans*.

### 2.3. CmCH1 Compromises Cell Wall Integrity of C. minitans

**Figure 4:**
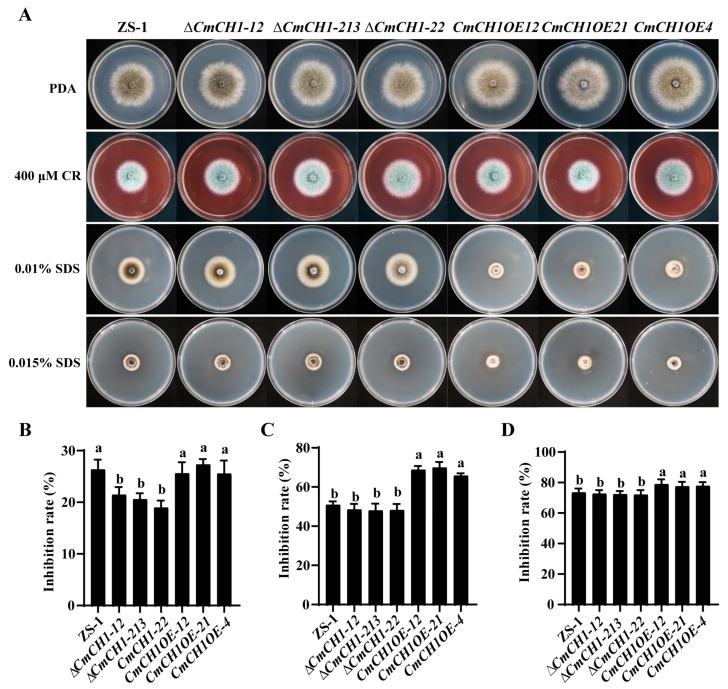
Sensitivity of *C. minitans* to Congo Red and SDS. (**A**) Colony morphology of *C. minitans* under different stresses (20 °C, 10 d). (**B**) Inhibition rate of *C. minitans* on PDA containing 400 μM CR. (**C**) Inhibition rate of *C. minitans* on PDA containing 0.01% SDS. (**D**) Inhibition rate of *C. minitans* on PDA containing 0.015% SDS. Different lowercase letters (a, b) indicate significant differences between strains. One-way ANOVA, *p* < 0.05, n = 8.

To determine whether *CmCH1* affects the cell wall of *C. minitans*, we evaluated the impact of abiotic stresses, CR and SDS, on cell wall integrity (Figure 4A). All *C. minitans* strains formed a colony with dense white mycelia on CR-containing PDA. Notably, *CmCH1* knockout mutants exhibited lower sensitivity compared to the wild-type strain ZS-1. The growth inhibition rates of three knockout mutants were 21.59 ± 1.26%, 20.42 ± 0.95%, and 18.96 ± 1.29%, whereas strain ZS-1 showed an inhibition rate of 26.30 ± 1.76% under 400 μM CR. In contrast, overexpression mutants displayed no significant difference in growth inhibition compared to the wild type (Figure 4B). When SDS was employed as a stressor, all strains showed significant growth suppression. On PDA containing 0.01% SDS, *CmCH1* knockout mutants exhibited no significant difference in the growth inhibition rate compared to the ZS-1 strain. However, the overexpression mutants demonstrated significantly enhanced growth suppression, with an average inhibition rate 33.97% higher than that of strain ZS-1 (Figure 4C). Similar trends were observed at 0.015% SDS, where overexpression mutants displayed significantly higher sensitivity than both the WT and knockout strains (Figure 4D). These results indicate that *CmCH1* compromises *C. minitans* cell wall integrity, likely due to altered chitin deposition in the cell wall caused by differential *CmCH1* expression.

To further evaluate the impact of *CmCH1* on cell wall integrity in *C. minitans*, we assessed protoplast release efficiency and conidial germination characteristics in *CmCH1* knockout and overexpression mutants. The protoplast released efficiency was quantified by counting the number of released protoplasts using a hemocytometer under a light microscope. The number of protoplasts released by both knockout and overexpression mutants showed no significant difference compared to strain ZS-1 but exhibited a significant difference between the two types of mutants. The germination pattern of conidia cultured for 24 h demonstrated results similar to those observed in the protoplast release capability (Table 1). The results demonstrate that the differential expression of *CmCH1* affected the cell wall integrity of *C. minitans*.

### 2.4. Simultaneous Deletion of CmCH1 and CmCH10 Leads to Growth Delay in C. minitans

Neither knockout nor overexpression of *CmCH1* significantly altered the mycoparasitic capability of *C. minitans*, a phenomenon potentially attributable to genetic redundancy. To investigate this further, we examined the expression patterns of other chitinase-encoding genes during the parasitism of *CmCH1* knockout mutant with *S. sclerotiorum*.

**Figure 5:**
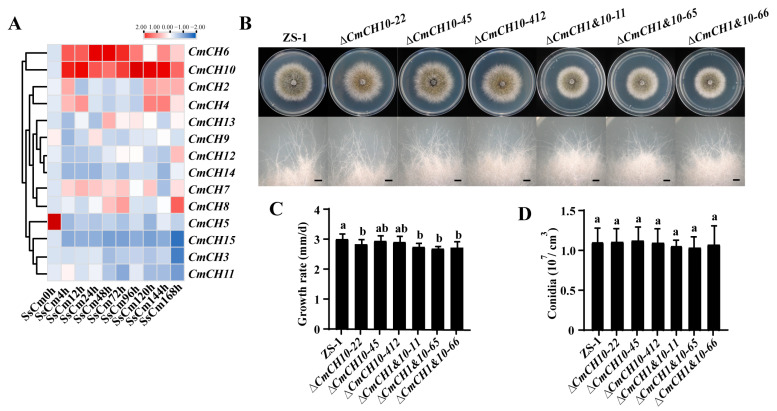
Biological characteristic of *C. minitans* strains. (**A**) The expression of the other fourteen chitinase genes during the interaction between *∆CmCH1-213* and *S. sclerotiorum.* (**B**) Colony morphology (20 °C, 10 d) and mycelial tips (20 °C, 5 d) of *C. minitans* cultured on PDA. Bars = 1 mm. (**C**) The growth rate of *C. minitans*; n = 10. (**D**) The conidial production of *CmCH10* and *CmCH1&10* mutants; n = 8. Different lowercase letters (a, b) indicate significant differences between strains. One-way ANOVA, *p* < 0.05.

During the mycoparasitic interaction, the expression profiles of chitinase-encoding genes could be categorized into three distinct categories. The first category included genes with no or reduced expression (*CmCH3*, *CmCH5*, *CmCH11*, and *CmCH15*). The second category consisted of genes with increased expression (*CmCH2*, *CmCH4*, *CmCH7*, *CmCH8*, *CmCH9*, *CmCH12*, *CmCH13*, and *CmCH14*). The final category, which warranted particular attention, included genes with significantly increased expression (*CmCH6* and *CmCH10*). Notably, the expression of *CmCH6* demonstrated a trend of initially increasing followed by decreasing in the interaction, while the expression of *CmCH10* showed a continuous upregulation trend throughout the interaction (Figure 5A). This suggests that *CmCH10* might play a role in the mycoparasitic process.

To elucidate the function of *CmCH10* in mycoparasitism, three double-knockout mutants (*∆CmCH1&10-11*, *∆CmCH1&10-65*, and *∆CmCH1&10-66*) were generated by disrupting *CmCH10* in *∆CmCH1-213* (Figure S3B). Parallel experiments created three *CmCH10* single-knockout mutants (*∆CmCH10-22*, *∆CmCH10-45*, and *∆CmCH10-412*) in the wild-type strain ZS-1 (Figure S3C).

The morphology of the colonies and mycelial tips was examined on PDA. *CmCH10* knockout mutants and *CmCH1&10* double-knockout mutants exhibited similar colony morphology and mycelial tips compared to the WT strain ZS-1 (Figure 5B). *CmCH1&10* double-knockout mutants exhibited growth rates of 2.74 ± 0.12 mm/d, 2.68 ± 0.07 mm/d, and 2.75 ± 0.15 mm/d, which were significantly slower than that of the WT strain ZS-1 (2.99 ± 0.17 mm/d). However, no statistically significant differences were detected in growth rates between the *CmCH10* single-knockout mutants and the strain ZS-1 (Figure 5C). Neither *CmCH10* knockout mutants nor *CmCH1&10* double-knockout mutants exhibited significant differences in conidiation compared to that of the WT strain ZS-1 (Figure 5D). The results indicate that *CmCH10* deletion may have little influence on *C. minitans*, while concurrent deletion of *CmCH1* and *CmCH10* reduced the growth of *C. minitans*.

### 2.5. Double Deletion of CmCH1 and CmCH10 Decreases Parasitic Ability of C. minitans

**Figure 6:**
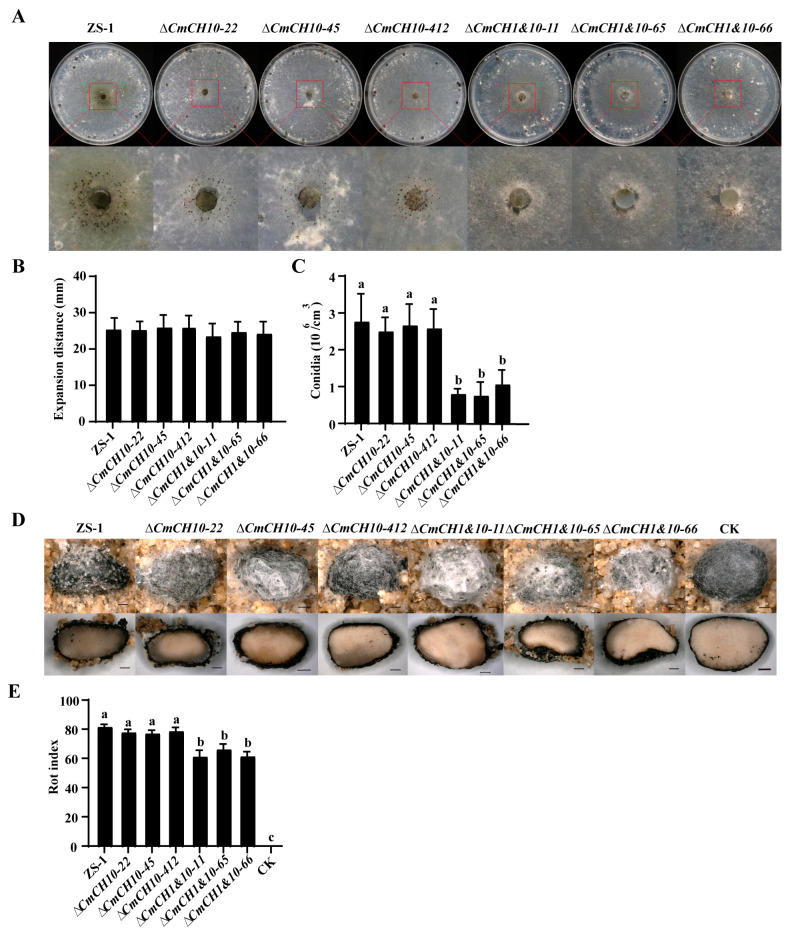
The parasitic ability of *CmCH10* and *CmCH1&10* knockout mutants against *S. sclerotiorum*. (**A**) The parasitism of *CmCH10* and *CmCH1&10* mutants on colony of 1980 (20 °C, 30 d). (**B**) The expansion distance of *CmCH10* and *CmCH1&10* mutants on colony of 1980 on water agar; n = 8. (**C**) The conidial production of *CmCH10* and *CmCH1&10* mutants on colony of 1980 on water agar; n = 16. (**D**) The surface and cross-section of sclerotia parasitized by *CmCH1* mutants. Bar = 1 mm. Sclerotia treated with sterile water were used as the negative control. (**E**) The rot index of sclerotia treated with conidia of *CmCH10* and *CmCH1&10* mutants. The sclerotia in the CK group showed no decay, resulting in a rot index of 0; n = 3. Different lowercase letters (a, b, and c) indicate significant differences between strains. One-way ANOVA, *p* < 0.05.

The parasitic capability of *CmCH1* and *CmCH1&10* mutants against *S. sclerotiorum* was assessed using the method above. The results showed that both *CmCH1* and *CmCH1&10* mutants could normally produce pycnidia and conidia on the colony of *S. sclerotiorum* (Figure S4A). On PDA, no significant difference was observed in the mycoparasitic ability of the mutants compared to the wild-type strain ZS-1 (Figure S4B). To eliminate the potential nutritional interference from PDA, water agar was used for co-culturing *C. minitans* with *S. sclerotiorum*. The results revealed that melanin produced by *CmCH10* knockout mutants was reduced, while *CmCH1&10* double mutants exhibited a decrease in pycnidia formation (Figure 6A). Although the mycelial growth of all *C. minitans* mutants on *S. sclerotiorum* showed no significant difference from strain ZS-1 (Figure 6B), the conidial production of *CmCH1&10* double mutants was significantly reduced (Figure 6C). To evaluate the sclerotial parasitism ability of the mutants, conidial infection assays were performed. All *C. minitans* mutants caused sclerotia rot and produced visible pycnidia (Figure 6D). The average rot indices of *CmCH10* knockout mutants and *CmCH1&10* double-knockout mutants were 77.57 ± 0.64 and 62.64 ± 2.30, respectively. Notably, the rot indices of sclerotia infected by the *CmCH1&10* double-knockout mutants were significantly lower than that of strain ZS-1 (81.38 ± 1.55) (Figure 6E). These results indicate that the double deletion of *CmCH1* and *CmCH10* weakened the parasitic ability of *C. minitans* against both the mycelia and sclerotia of *S. sclerotiorum*.

### 2.6. Double Deletion of CmCH1 and CmCH10 Enhances Tolerance of C. minitans to Abiotic Stress

**Figure 7:**
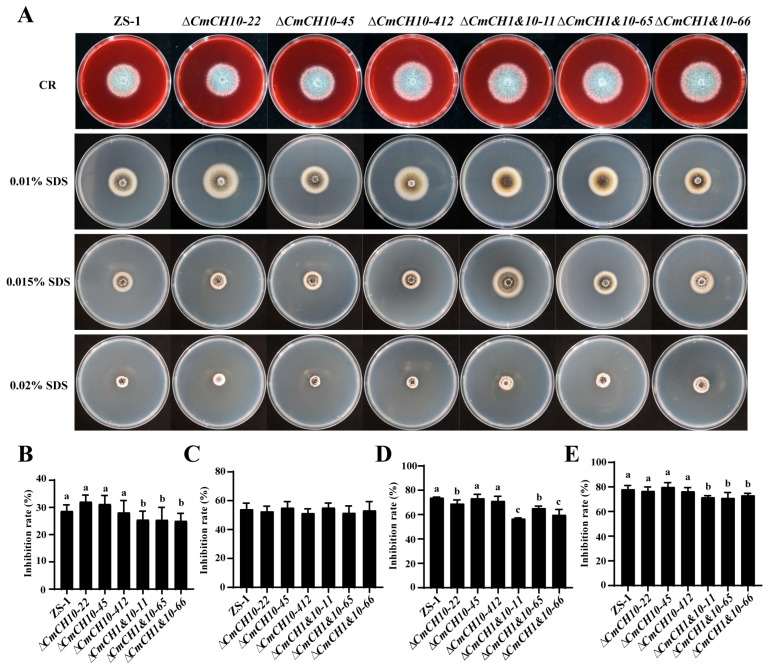
Tolerance of *CmCH10* and *CmCH1&10* knockout mutants to abiotic stresses. (**A**) The colony morphology of *CmCH10* and *CmCH1&10* knockout mutants cultured on PDA supplemented with 400 µM CR and SDS (20 °C, 10 d). (**B**) The growth inhibition rate of *C. minitans* cultured on PDA supplemented with 400 µM CR, n = 8. (**C**–**E**) The growth inhibition rate of *C. minitans* cultured on PDA supplemented with 0.01%, 0.015%, and 0.02% SDS (*w*/*v*), n = 8. Different lowercase letters (a, b, and c) indicate significant differences between strains. One-way ANOVA, *p* < 0.05.

The sensitivity of *CmCH10* and *CmCH1&10* knockout mutants to CR and SDS was examined. All *C. minitans* strains were capable of growing on PDA containing 400 µM CR, developing a colony with dense white mycelial growth. However, when cultured on PDA containing 0.01%, 0.015%, and 0.02% SDS (*w*/*v*), significant growth inhibition was observed (Figure 7A). On PDA containing 400 μM CR, the growth inhibition rates of three *CmCH10* knockout mutants were 32.05 ± 2.36%, 31.20 ± 2.97%, and 28.12 ± 4.14%, showing no significant difference with that of strain ZS-1 (28.66 ± 2.15%). In contrast, three *CmCH1&10* double-knockout mutants exhibited significantly different growth inhibition rates by 25.45 ± 2.96%, 25.39 ± 4.31%, and 25.02 ± 2.61% (Figure 7B). Under 0.01% SDS stress conditions, no statistically significant differences in growth inhibition were detected between all knockout mutants and the ZS-1 strain (Figure 7C). However, at higher SDS concentrations (0.015% and 0.02%), *CmCH1&10* double-knockout mutants exhibited significantly lower sensitivity compared to *CmCH10* knockout mutants and strain ZS-1 (Figure 7D,E). These results indicate that the double deletion of *CmCH1* and *CmCH10* enhances cell wall integrity and resistance to abiotic stress in *C. minitans*.

The protoplast release capacity and conidial germination rate of *CmCH10* and *CmCH1&10* mutants were evaluated. The results demonstrated that under enzymatic digestion, the number of protoplasts released by the three double-knockout mutants at 1 h were 0.83 ± 0.75, 0.67 ± 0.55, and 0.75 ± 0.69 × 10^5^/cm^3^. These values were far lower than those of the *CmCH10* knockout mutants and strain ZS-1, which were 2.92 ± 1.30, 3.25 ± 1.18, 2.47 ± 1.72 × 10^5^/cm^2^, and 3.00 ± 0.65 × 10^5^/cm^3^, respectively. No significant difference was observed between the three *CmCH10* knockout mutants and strain ZS-1 (Table 2). A further analysis of conidial germination revealed that the *CmCH1&10* double-knockout mutants exhibited a significant 26.48% reduction in germination rate compared to strain ZS-1 (Table 2). Staining with 10 μg/mL WGA488 revealed enhanced chitin deposition in the cell walls of double-knockout mutants compared to strain ZS-1 (Figure S5). The results indicate that *CmCH1&10* double-knockout mutants possess a more solid cell wall, thereby enhancing the stress resistance of *C. minitans*. *CmCH1* and *CmCH10* might function in cell wall remodeling and chitin deposition processes.

## 3. Discussion

Chitin, the second most abundant natural polymer in nature, is degraded by chitinases, a class of enzymes widely distributed across organisms. In human, chitinase expression correlates with diseases; for example, chitinase-like protein YKL-40 is highly expressed in severe asthma patients, linking its activity to disease severity [30]. In birds, chitinases in the digestive tract aid in digesting chitin-containing insects [31]. Arthropods rely on chitin as a key component of their exoskeletons, with chitinases facilitating chitin remodeling during molting and supporting digestion in detritivores or predators [32,33]. Plants, despite having low chitinase expression, utilize these enzymes as direct defenses against pathogens [34,35]. Intriguingly, oomycetes like *Phytophthora*, which lack chitin, encode multiple chitinase genes with expression varying by species: hyperparasitic species exhibit high expression, while pathogenic species show minimal expression, suggesting a role in hyperparasitism [36]. *Autographa californica* nuclear polyhedrosis virus (AcMNPV) harbors a functional chitinase gene (*chiA*), likely acquired via horizontal gene transfer from bacteria, which is expressed during late viral replication [37]. In fungi, chitinases are indispensable, governing critical processes such as cell wall dynamics and hyphal growth. These diverse roles underscore the evolutionary and ecological significance of chitinases across life forms.

The chitinase genes exhibit characteristics of diversity. Phylogenetic analysis of GH18 family members classifies them into three evolutionarily distinct subgroups, namely Subgroup A, Subgroup B, and Subgroup C [38]. The three subgroups exhibit distinct structural architectures. Subgroup A members possess solely a catalytic domain (GH18), while Subgroup B members contain C-terminal carbohydrate-binding modules (CBMs). In contrast, Subgroup C displays a structure fundamentally divergent from both A and B, typically assembling into large macromolecular complexes with intricate structural organization [38,39,40]. Fifteen chitinases in *C. minitans* can also be structurally classified. For instance, CmCH1, CmCH3, CmCH4, CmCH8, CmCH11, CmCH14, and CmCH15 may be classified into Subgroup A; CmCH2, CmCH7, and CmCH9 may belong to Subgroup B; while CmCH5, CmCH6, CmCH10, CmCH12, and CmCH13 may be categorized under Subgroup C (Figure S1A). This classification of chitinases in *C. minitans* is currently speculative and requires experimental verification, and such clarification would facilitate the functional characterization of these enzymes. Structural divergence suggests that chitinases fulfill distinct functional roles during differentiation. Furthermore, nine of these fifteen chitinases are secretory proteins (Figure S1B), indicating their potential to participate in the mycoparasitic activity of *C. minitans*.

The expression of chitinase genes is regulated under specific induction conditions or developmental stages. *Ech42* in *T. atroviride* participates in both its own cell wall remodeling process and mycoparasitism. In *T. atroviride*, the expression of *chit33* and *ech30* is significantly induced by chitin-related components of the host cell wall while being negatively regulated by metabolites such as glucose. Additionally, the expression of chitinase genes differs between normal cultivation conditions and nutrient deprivation [39,41]. In *C. minitans*, chitinase gene expression varies across developmental stages. Specifically, *CmCH3*, *CmCH7*, and *CmCH12* show upregulated expression during the early mycoparasitic stage and mycelial development stage, while *CmCH1* and *CmCH9* exhibit increased expression at the late mycoparasitic stage and conidial formation stage (Figure 2). Functional characterization of multiple chitinase genes (*CmCH1*, *CmCH4*, *CmCH8*, *CmCH9*, and *CmCH10*; mutant data for genes other than *CmCH1* and *CmCH10* remain unpublished) revealed that individual gene knockouts did not significantly alter fungal phenotypes—even for *CmCH1*, a well-established marker gene in mycoparasitism. Genetic redundancy may compensate for the phenotypic changes in single-gene mutants, a phenomenon also documented in other studies [42,43]. Similar studies suggest that fungal cell wall degradation and remodeling are complex processes involving coordinated regulation by multiple enzymes and structural proteins and that phenotypic changes generally require the study of multiple deletion mutants [44,45,46]. Therefore, we simultaneously knocked out multiple chitinase genes to further explore their functions. The results showed that the double-knockout mutants (*CmCH1* and *CmCH10*) showed significant differences in cell wall integrity and conidial production ability compared to the WT, which may be the reason for the reduced ability to parasitize *S. sclerotiorum*.

Chitin typically resides in the innermost layer of the cell wall of filamentous fungi and is encased by various substances such as proteins, mannan, and β-1,3-glucan [41]. Therefore, under intact cellular conditions, chitinases cannot directly access and degrade chitin. The determinant for chitinase from mycoparasitic fungi to degrade chitin in host cells is the accessibility of its chitinase to chitin. Both mycoparasitic fungi and their fungal hosts are filamentous fungi sharing similar core cell wall components. This similarity raises the question of how mycoparasitic fungi distinguish the “self” from the “non-self” via its chitinases. In addition, fungi have evolved different strategies to protect themselves. Research has shown that *Cladosporium fulvum* avirulent protein Avr4 can protect its cell wall chitin from damage by chitinases [47]. *T. harzianum* cell wall protein QID74 contributes to maintaining the integrity of its own cell wall [48]. However, some studies indicate that chitinases from *Trichoderma* species degrade fungal cell walls indiscriminately, suggesting the lack of intrinsic specificity for “self” vs. “non-self” [40]. To date, no studies have delineated strict functional specialization among mycoparasitic chitinases. Future work using advanced imaging techniques to observe mycoparasite–host interactions may reveal the mechanism by which chitinases recognize the “self” from the “non-self”.

## 4. Materials and Methods

### 4.1. Strain and Growth Conditions

The wild-type (WT) strain ZS-1 of *C. minitans* (GenBank accession GCA_009707825.1) was originally isolated from rotting sclerotia in Zhushan County, Hubei Province, China [49]. The wild-type strain 1980 of *S. sclerotiorum* (GenBank accession GCA_000146945.2) served as the host fungus to evaluate the mycoparasitism of *C. minitans*. All fungal strains were cultured on potato dextrose agar plate (PDA) medium at 20 °C and stored at 4 °C. For molecular cloning procedures, *Escherichia coli* strain DH5α was cultured on Luria–Bertani (LB) at 37 °C [11,49] and *Agrobacterium tumefaciens* EHA106 at 28 °C. The yeast strain YTK12, for verifying the function of the signal peptide, was cultured on Yeast Extract Peptone Dextrose (YPD) at 28 °C [50].

### 4.2. Sequence Analysis

The putative chitinase-encoding gene sequences of *C. minitans* were initially retrieved from a local database, with their homologous sequences subsequently identified from the National Center for Biotechnology Information (NCBI) GenBank database. Conserved domains of the GH18 family were predicted using the NCBI Conserved Domain Search Tools. Concurrently, CD Search was analyzed using the conserved domain database (CDD) v3.21-62456 PSSM (https://www.ncbi.nlm.nih.gov/Structure/cdd/wrpsb.cgi) [accessed on 2 September 2025] to characterize conserved domains within the complete amino acid sequences. Phylogenetic analysis based on amino acid sequences was performed using the neighbor-joining (NJ) method and tested with a bootstrap of 1000 replicates to ascertain the reliability of a given branch pattern in MEGA 6 (v 6.06) [51]. Subsequently, multiple sequence alignment of conservative domains was performed using ClustalW with the default program in Jalview software (v 2.11.1.0), while sequence conservation patterns were visualized through sequence logos generated by weblogo (http://weblogo.berkeley.edu/logo.cgi) [accessed on 2 September 2025] [52].

### 4.3. Yeast Secretion Trap Screen Assay

The signal peptides of putative chitinase-encoding genes were predicted. Subsequently, the predicted signal peptide sequences were fused to the N-terminus of the secretion-defective invertase gene (*suc2*) in the vector pSUC2 (Figure S6A) and then transformed into the yeast strain YTK12 using the LiAc/SS carrier DNA/PEG method [50,53]. Yeast mutants were screened on YPRAA (10 g/L yeast extract, 20 g/L peptone, 20 g/L raffinose, 2 mg/L antimycin A, and 2% agar). Only mutants with secretion activity were able to grow on YPRAA. The secretory invertase activity of yeast mutants was assessed using the 2,3,5-triphenyltetrazolium chloride (TTC) assay. Briefly, mutants were incubated in a 10% sucrose solution at 30 °C for 35 min, followed by centrifugation to collect the supernatant. After adding TTC reagent for a final concentration of 0.1%, the reaction mixture was incubated at room temperature for 5 min to observe the color change in the test tube; the Avrb mutant was used as the positive control, the pUSC2 mutant was used as the negative control, and the YTK12 strain was used as the blank control. A positive reaction would change from colorless to dark red.

### 4.4. Gene Deletion and Overexpression

The deletion of the *CmCH1* gene was conducted using the split marker system [54]. The 5′ and 3′ flanking fragments of *CmCH1* CDS were amplified from the genomic DNA of the wild-type *C. minitans* strain ZS-1. Two truncated fragments of the hygromycin-resistant gene were amplified from plasmid pUCH18 (Figure S6B). Subsequently, the 5′ and 3′ flanking fragments of the *CmCH1* CDS were individually ligated with their corresponding truncated resistance gene fragments. The two fusion fragments were then amplified and utilized for protoplast transformation, as described by Kohn [55]. Briefly, *C. minitans* hypha were treated with 0.015 g/mL lysing enzymes from *T. harzianum* (L1412, Sigma, St. Louis, MO, USA) at 30 °C for 2 h. The protoplasts were then harvested by filtration followed by centrifugation at 3000× *g* for 10 min. The two fusion fragments were introduced into the protoplasts via PEG-mediated transformation. The knockout mutants were selected on plates with hygromycin and confirmed through PCR.

For *CmCH1* overexpression, *CmCH1*-GFP was amplified and cloned into plasmid pCETNS (Figure S6C). The recombinant plasmid was then transformed into *A. tumefaciens* EHA105 via electroporation and used to transform the conidia of strain ZS-1. Mutants were selected on plates containing G418 and confirmed through PCR and RT-PCR.

The same knockout strategy employed for *CmCH1* was applied to disrupt *CmCH10*, except that the hygromycin-resistant gene was replaced with a bialaphos-resistant gene. The vector used was pBAR (Figure S6D). The putative knockout mutants of *CmCH10* were selected on plates containing Basta and confirmed by PCR. Information on the gene-specific primers is listed in Table S1.

### 4.5. Phenotypic Analysis of Mutants

To evaluate the growth rates of both the WT and mutant strains, mycelial plugs (5 mm in diameter) were aseptically collected from the actively growing margin of colonies and inoculated onto PDA plates. After incubation for 5 days, colony diameter was measured, and the hyphal tips were observed under a microscope. All *C. minitans* strains were grown on PDA for 15 days, and then the amounts of conidia were recorded. For conidial germination, conidia of 1 × 10^5^/mL in PDB were incubated with shaking (150 rpm) for 24 h, and then germination rates were determined. For the protoplast release assay, protoplasts were prepared as described above with lysing enzyme treatment for 1 h and were subsequently quantified by hemocytometer counting under a microscope.

To detect stress tolerance, mycelial plugs (5 mm in diameter) were incubated on PDA containing stress factors, including sodium dodecyl sulfate (SDS, 0.01%, 0.015%, and 0.02%, *w*/*v*) and Congo red (400 μM). Colony morphology was documented at 10 dpi, and the hyphal growth inhibition rate was calculated using the following formula:

Inhibition rate (%) = (1 − Colony diameter (stress)/Colony diameter (control)) × 100

For each assay, three independent biological replicates were performed, each containing three technical repetitions.

### 4.6. Mycoparasitic Ability Assay

To evaluate the mycoparasitic ability of *C. minitans* against the hypha of *S. sclerotiorum*, two distinct experimental strategies were employed. All cultures were maintained at 20 °C in complete darkness with 50% relative humidity. A PDA plate confrontation assay was performed as described by Zeng [25]. To eliminate the potential confounding effects of nutrient availability from the culture medium, the mycoparasitic capability of *C. minitans* against *S. sclerotiorum* was evaluated on water agar (WA). Briefly, mycelial plugs (5 mm in diameter) of strain 1980 were excised from colony margins and inoculated onto cellophane-overlaid PDA plates (20 mL medium per plate). After the hypha fully colonized the cellophane, the central *S. sclerotiorum* plug was carefully removed, and the cellophane sheet was transferred onto a WA plate (20 mL). Fresh mycelial plugs (5 mm in diameter) of *C. minitans* strains were then placed at the vacant central region of the cellophane. The mycoparasitic ability was examined 20 days after incubation. Parasitized sclerotia were fixed with tweezers and divided into cross-sections using a blade. The mycoparasitic ability of *C. minitans* against the sclerotia of *S. sclerotiorum* was investigated according to the method by Cheng [49].

For quantitative analysis, mixed hypha of the same area were homogenized and washed, and conidial concentration was determined. Data from three technical replicates per strain were averaged, with three independent biological replicates performed.

### 4.7. Sample Collection, RNA Extraction, and Quantitative Real-Time PCR Analysis

To elucidate the gene expression dynamics during the parasitic interaction between *C. minitans* and *S. sclerotiorum*, hyphal samples were collected at ten time points throughout the parasitism process. A conidial suspension of *C. minitans* (1 × 10^6^ conidia/mL) was inoculated into PDB and incubated with shaking (150 rpm) in darkness for 36 h. Germinated conidia were collected by centrifuging (5000 rpm, 5 min), washed three times with sterile water, and resuspended. Fresh mycelial plugs of the strain 1980 were pre-inoculated on cellophane-overlaid PDA for 36 h. The mycelium-covered cellophane was transferred onto WA so that germinated conidia of *C. minitans* could be evenly spread over the colony of strain 1980. Mixed hyphal samples were collected at 0, 4, 12, 24, 48, 72, 96, 120, 144, and 168 hpi and stored at −80 °C for RNA extraction. The 0 hpi served as the control. Three biological replicates were analyzed per time point.

Total RNA was extracted with the RNA reagent (Diyue Biotechnology, Wuhan, China), and first-strand cDNA was synthesized using the *TransScript*^®^ One-Step gDNA Removal and cDNA Synthesis SuperMix Kit (TransGen Biotech, Beijing, China; Catalog# AT311-02).

Quantitative real-time PCR (qRT-PCR) was conducted using *PerfectStart*^®^ Green qPCR SuperMix (TransGen Biotech, Beijing, China; Catalog# AQ601-01-V2) on the Bio-Rad CFX Real-Time System (Bio-Rad, Berkeley, CA, USA). The specific operation was performed as described by Zhao [56]. The total cDNA abundance in the samples was standardized against the *C. minitans Actin* gene. All samples were subjected to three technical replicates. The transcript levels were calculated by the 2^−∆∆Ct^ method. The results were presented using a heat map. The heatmap was made by TBtools (v 2.3.09). The primers were designed using Primer Premier 5.0 (v 5.00), and the specific primer sequences are listed in Table S1.

### 4.8. Transcriptomic Analysis

The mixed mycelia of *C. minitans* and *S. sclerotiorum* were collected at the time points of 0 h, 4 h, 12 h, 24 h, 48 h, 72 h, 96 h, 120 h, 144 h, and 168 h, and then RNA was extracted and sent to the company for sequencing. After the RNA samples passed the quality inspection, library construction was carried out. The libraries that passed the quality inspection were sequenced on an Illumina NovaSeq 6000 platform (Illumina, San Diego, CA, USA) in PE150 (paired-end) mode by Biomarker Technologies Corporation (Beijing, China). The raw data obtained after sequencing were processed to remove the reads containing adapters and those of low quality. After a series of quality control steps, high-quality clean data were obtained. The clean data were then aligned with the specified fungal reference genome. In this paper, StringTie was used to perform normalization by means of the maximum flow algorithm and using FPKM (fragments per kilobase of transcript per million fragments mapped) as an indicator to measure the expression levels of transcripts or genes. Using the DESeq2_edgeR package on the BioCloud platform (https://international.biocloud.net/zh/dashboard) [accessed on 2 September 2025] with stringent thresholds (fold change ≥ 2.0 and *p*-value ≤ 0.01), transcriptomic profiling was conducted to investigate the mycoparasitic interaction between *C. minitans* and *S. sclerotiorum*.

### 4.9. Microscopic Analysis

To observe chitin distribution on the cell walls of mutant strains, mycelia were stained with 10 μg/mL wheat germ agglutinin conjugated to Alexa Fluor 488 (WGA488, Invitrogen, Cat. No. W11261) and incubated at room temperature for 30 min, followed by removal of the staining solution and three washes with phosphate-buffered saline (PBS). The samples were then examined under a confocal microscope (Leica SP8; Leica Microsystems, Mannheim, Germany) with an excitation wavelength of 495 nm and emission wavelength of 519 nm.

### 4.10. Statistical Analysis

Transcriptome data were analyzed by dividing the FPKM (millions of fragments per kilobase) value of genes involved in the interaction between *C. minitans* and *S. sclerotiorum* or during the growth and development stages of *C. minitans* [26] by the average of all values, and the ratio is converted to log2. The results were represented as heatmap made by TBtools (v 2.3.09). Statistical analyses were performed using the one-way ANOVA method with GraphPad Prism 8.0 (v 8.0.2) with a significance level set to *p* < 0.05. Prior to the ANOVA, the normality of data distribution was verified using the Shapiro–Wilk test, and the homogeneity of variances was confirmed using Bartlett’s test. Among the data, there was no significant correlation. All experiments were repeated independently at least three times, and the exact number of biological replicates (n) for each experiment is provided in the respective figure legends and tables.

## Figures and Tables

**Table 1:** Protoplasts released and conidia germination of *C. minitans*.

Strain	Protoplasts (10^5^/cm^3^)	Conidial Germination (%)
ZS-1	3.10 ± 1.02 ab	61.33 ± 0.94 ab
*∆CmCH1-12*	2.40 ± 1.02 b	57.66 ± 1.25 b
*∆CmCH1-213*	2.50 ± 0.89 b	58.00 ± 0.82 b
*∆CmCH1-22*	2.30 ± 0.40 b	58.00 ± 1.63 b
CmCH1OE-12	4.90 ± 1.11 a	64.00 ± 0.82 a
CmCH1OE-21	4.50 ± 0.32 a	63.67 ± 1.25 a
CmCH1OE-4	4.80 ± 0.68 a	64.67 ± 0.47 a

The values represent means derived from three independent replicates, and the error bars indicate ± SD of the mean. Different lowercase letters (a, b) represent significant differences between strains. One-way ANOVA, *p* < 0.05, n ≥ 3.

**Table 2:** Protoplasts released and conidia germination of *C. minitans* strains.

Strain	Protoplasts (10^5^/cm^3^)	Conidial Germination (%)
ZS-1	3.00 ± 0.65 a	69.67 ± 4.82 a
*∆CmCH10-22*	2.92 ± 1.30 a	63.00 ± 12.42 a
*∆CmCH10-45*	3.25 ± 1.18 a	62.67 ± 6.50 a
*∆CmCH10-412*	2.47 ± 1.72 a	60.67 ± 9.91 a
*∆CmCH1&10-11*	0.83 ± 0.75 b	47.67 ± 9.55 b
*∆CmCH1&10-65*	0.67 ± 0.55 b	52.33 ± 5.09 b
*∆CmCH1&10-66*	0.75 ± 0.69 b	53.67 ± 3.73 b

The values represent means derived from three independent replicates, and the error bars indicate ± SD of the mean. Different lowercase letters (a, b) represent significant differences between strains One-way ANOVA, *p* < 0.05, n = 6.

## Data Availability

The original contributions presented in this study are included in the article. Further inquiries can be directed to the corresponding author.

## Supplementary Materials

The following supporting information can be downloaded at: https://www.mdpi.com/article/10.3390/ijms26178706/s1.

## References

[1] Boland G.J., Hall R. (1994). Index of plant hosts of *Sclerotinia sclerotiorum*. Can. J. Plant Pathol..

[2] Singh J., Yadav P., Budhlakoti N., Mishra D.C., Bhardwaj N.R., Rao M., Sharma P., Gupta N.C. (2024). Exploration of the *Sclerotinia sclerotiorum*-Brassica pathosystem: Advances and perspectives in omics studies. Mol. Biol. Rep..

[3] Shang Q., Jiang D., Xie J., Cheng J., Xiao X. (2024). The schizotrophic lifestyle of *Sclerotinia sclerotiorum*. Mol. Plant Pathol..

[4] Liang X., Rollins J.A. (2018). Mechanisms of broad host range necrotrophic pathogenesis in *Sclerotinia sclerotiorum*. Phytopathology.

[5] Campbell W.A. (1947). A new species of *Coniothyrium* parasitic on sclerotia. Mycologia.

[6] Huang H.C., Erickson R.S. (2010). Factors affecting biological control of *Sclerotinia sclerotiorum* by fungal antagonists. J. Phytopathol..

[7] Huang H.C. (1977). Importance of *Coniothyrium minitans* in survival of sclerotia of *Sclerotinia sclerotiorum* in wilted sunflower. Can. J. Bot..

[8] McQuilken M.P., Chalton D. (2009). Potential for biocontrol of sclerotinia rot of carrot with foliar sprays of Contans? WG (*Coniothyrium minitans*). Biocontrol Sci. Technol..

[9] Sun X., Zhao Y., Jia J., Xie J., Cheng J., Liu H., Jiang D., Fu Y. (2017). Uninterrupted expression of *CmSIT1* in a sclerotial parasite *Coniothyrium minitans* leads to reduced growth and enhanced antifungal ability. Front. Microbiol..

[10] Yang R., Han Y.C., Li G.Q., Jiang D.H., Huang H.C. (2007). Suppression of *Sclerotinia sclerotiorum* by antifungal substances produced by the mycoparasite *Coniothyrium minitans*. Eur. J. Plant Pathol..

[11] Zeng F., Gong X., Hamid M.I., Fu Y., Jiatao X., Cheng J., Li G., Jiang D. (2012). A fungal cell wall integrity-associated MAP kinase cascade in *Coniothyrium minitans* is required for conidiation and mycoparasitism. Fungal Genet. Biol..

[12] Wei W., Zhu W., Cheng J., Xie J., Jiang D., Li G., Chen W., Fu Y. (2016). Nox Complex signal and MAPK cascade pathway are cross-linked and essential for pathogenicity and conidiation of mycoparasite *Coniothyrium minitans*. Sci. Rep..

[13] Jones D., Watson D. (1969). Parasitism and lysis by soil fungi of *Sclerotinia sclerotiorum* (Lib.) de Bary, a phytopathogenic fungus. Nature.

[14] Giczey G.B., Kerényi Z.N., Fülöp L.S., Hornok L.S. (2001). Expression of cmg1, an exo-β-1,3-Glucanase Gene from *Coniothyrium minitans*, Increases during Sclerotial Parasitism. Appl. Environ. Microbiol..

[15] Yang X., Zhao H., Luo C., Du L., Cheng J., Xie J., Jiang D., Fu Y. (2020). CmAim24 is essential for mitochondrial morphology, conidiogenesis, and mycoparasitism in *Coniothyrium minitans*. Appl. Environ. Microbiol..

[16] Rogers C.W., Challen M.P., Muthumeenakshi S., Sreenivasaprasad S., Whipps J.M. (2008). Disruption of the *Coniothyrium minitans* PIF1 DNA helicase gene impairs growth and capacity for sclerotial mycoparasitism. Microbiology.

[17] Singh G., Bhalla A., Bhatti J.S., Ch S., Rajput A., Abdullah A., Andrabi W., Kaur P. (2014). Potential of chitinases as a biopesticide against agriculturally harmful fungi and insects. Res. Rev..

[18] Huang Q.-S., Xie X.-L., Liang G., Gong F., Wang Y., Wei X.-Q., Wang Q., Ji Z.-L., Chen Q.-X. (2012). The GH18 family of chitinases: Their domain architectures, functions and evolutions. Glycobiology.

[19] Kubicek C.P., Herrera-Estrella A., Seidl-Seiboth V., Martinez D.A., Druzhinina I.S., Thon M., Zeilinger S., Casas-Flores S., Horwitz B.A., Mukherjee P.K. (2011). Comparative genome sequence analysis underscores mycoparasitism as the ancestral life style of *Trichoderma*. Genome Biol..

[20] Ulhoa C.J., Peberdy J.F. (1991). Regulation of chitinase synthesis in *Trichoderma harzianum*. J. Gen. Microbiol..

[21] García I., Lora J.M., Cruz J.d.l., Benítez T., Pintor-Toro J.A. (1994). Cloning and characterization of a chitinase (CHIT42) cDNA from the mycoparasitic fungus *Trichoderma harzianum*. Curr. Genet..

[22] Haran S., Schickler H., Oppenheim A., Chet I. (1995). New components of the chitinolytic system of *Trichoderma harzianum*. Mycol. Res..

[23] Dana M.D.L.M., Limón M.C., Mejías R., Mach R.L., Kubicek C.P. (2001). Regulation of chitinase 33 (*chit33*) gene expression in *Trichoderma harzianum*. Curr. Genet..

[24] Carsolio C., Gutierrez A., Jimenez B., Van Montagu M., Herrera-Estrella A. (1994). Characterization of *ech-42*, a *Trichoderma harzianum* endochitinase gene expressed during mycoparasitism. Proc. Natl. Acad. Sci. USA.

[25] Zeng L., Zhang J., Han Y.-C., Yang L., Wu M.-d., Jiang D.-H., Chen W., Li G.-Q. (2014). Degradation of oxalic acid by the mycoparasite *Coniothyrium minitans* plays an important role in interacting with *Sclerotinia sclerotiorum*. Environ. Microbiol..

[26] Zhao H., Zhou T., Xie J., Cheng J., Chen T., Jiang D., Fu Y. (2020). Mycoparasitism illuminated by genome and transcriptome sequencing of *Coniothyrium minitans*, an important biocontrol fungus of the plant pathogen *Sclerotinia sclerotiorum*. Microb. Genom..

[27] Abeles F.B., Bosshart R.P., Forrence L.E., Habig W.H. (1971). Preparation and purification of glucanase and chitinase from bean leaves. Plant Physiol..

[28] Zhao Y., Li C., Chen X., Cheng J., Xie J., Lin y., Fu Y., Jiang D., Chen T. (2023). Overexpression of chitinase PbChia1 from *Plasmodiophora brassicae* improves broad-spectrum disease resistance of *Arabidopsis*. Virulence.

[29] Yang X., Yang J., Li H., Niu L., Xing G., Zhang Y., Xu W., Zhao Q., Li Q., Dong Y. (2020). Overexpression of the chitinase gene *CmCH1* from *Coniothyrium minitans* renders enhanced resistance to *Sclerotinia sclerotiorum* in soybean. Transgenic Res..

[30] Chupp G., Lee C.-M., Jarjour N.N., Shim Y.M., Holm C., He S., Dziura J., Reed J.L., Coyle A.J., Kiener P.A. (2007). A chitinase-like protein in the lung and circulation of patients with severe asthma. N. Engl. J. Med..

[31] Chen Y., Zhao H. (2019). Evolution of digestive enzymes and dietary diversification in birds. PeerJ.

[32] Merzendorfer H., Zimoch L. (2003). Chitin metabolism in insects: Structure, function and regulation of chitin synthases and chitinases. J. Exp. Biol..

[33] Gama M.d.V.F., Moraes C.S., Gomes B., Díaz-Albiter H., Mesquita R.D., Seabra-Junior E.S., Azambuja P., Garcia E.d.S., Genta F.A. (2022). Structure and expression of Rhodnius prolixus GH18 chitinases and chitinase-like proteins: Characterization of the physiological role of *RpCht7*, a gene from subgroup VIII, in vector fitness and reproduction. Front. Physiol..

[34] Boller T., Gehri A., Mauch F., Vögeli U. (1983). Chitinase in bean leaves: Induction by ethylene, purification, properties, and possible function. Planta.

[35] Schlumbaum A., Mauch F., Vögeli U., Boller T. (1986). Plant chitinases are potent inhibitors of fungal growth. Nature.

[36] Liang D., Andersen C.B., Vetukuri R.R., Dou D., Grenville-Briggs L.J. (2020). Horizontal gene transfer and tandem duplication shape the unique CAZyme complement of the mycoparasitic oomycetes *Pythium oligandrum* and *Pythium periplocum*. Front. Microbiol..

[37] Hawtin R.E., Arnold K., Ayres M.D., Zanotto P.M.d.A., Howard S.C., Gooday G.W., Chappell L.H., Kitts P.A., King L.A., Possee R.D. (1995). Identification and preliminary characterization of a chitinase gene in the *Autographa californica* nuclear polyhedrosis virus genome. Virology.

[38] Seidl V., Huemer B., Seiboth B., Kubicek C.P. (2005). A complete survey of *Trichoderma* chitinases reveals three distinct subgroups of family 18 chitinases. FEBS J..

[39] Seidl V. (2008). Chitinases of filamentous fungi: A large group of diverse proteins with multiple physiological functions. Fungal Biol. Rev..

[40] Gruber S., Seidl-Seiboth V. (2012). Self versus non-self: Fungal cell wall degradation in *Trichoderma*. Microbiology.

[41] Hartl L., Zach S., Seidl-Seiboth V. (2011). Fungal chitinases: Diversity, mechanistic properties and biotechnological potential. Appl. Microbiol. Biotechnol..

[42] Reichard U., Hung C.-Y., Thomas P.W., Cole G.T. (2000). Disruption of the gene which encodes a serodiagnostic antigen and chitinase of the human fungal pathogen *Coccidioides immitis*. Infect. Immun..

[43] Jaques A.K., Fukamizo T., Hall D., Barton R.C., Escott G.M., Parkinson T., Hitchcock C.A., Adams D.J. (2003). Disruption of the gene encoding the ChiB1 chitinase of *Aspergillus* fumigatus and characterization of a recombinant gene product. Microbiology.

[44] Dünkler A., Walther A., Specht C.A., Wendland J. (2005). *Candida albicans* CHT3 encodes the functional homolog of the Cts1 chitinase of *Saccharomyces cerevisiae*. Fungal Genet. Biol..

[45] Kämper J., Kahmann R., Bölker M., Ma L.J., Brefort T., Saville B.J., Banuett F., Kronstad J.W., Gold S.E., Müller O. (2006). Insights from the genome of the biotrophic fungal plant pathogen *Ustilago maydis*. Nature.

[46] López-Mondéjar R., Catalano V., Kubicek C.P., Seidl V. (2009). The beta-N-acetylglucosaminidases NAG1 and NAG2 are essential for growth of *Trichoderma atroviride* on chitin. FEBS J..

[47] van den Burg H.A., Harrison S.J., Joosten M.H.A.J., Vervoort J., de Wit P.J.G.M. (2006). *Cladosporium fulvum* Avr4 protects fungal cell walls against hydrolysis by plant chitinases accumulating during infection. Mol. Plant-Microbe Interact..

[48] Rosado I.V., Rey M., Codón A.C., Govantes J., Moreno-Mateos M.A., Benítez T. (2007). QID74 Cell wall protein of *Trichoderma harzianum* is involved in cell protection and adherence to hydrophobic surfaces. Fungal Genet. Biol..

[49] Cheng J., Jiang D., Yi X., Fu Y., Li G., Whipps J.M. (2003). Production, survival and efficacy of *Coniothyrium minitans* conidia produced in shaken liquid culture. FEMS Microbiol. Lett..

[50] Gietz R.D., Schiestl R.H. (2007). Large-scale high-efficiency yeast transformation using the LiAc/SS carrier DNA/PEG method. Nat. Protoc..

[51] Tamura K., Stecher G., Peterson D.S., Filipski A., Kumar S. (2013). MEGA6: Molecular evolutionary genetics analysis version 6.0. Mol. Biol. Evol..

[52] Waterhouse A.M., Procter J.B., Martin D.M., Clamp M., Barton G.J. (2009). Jalview Version 2--a multiple sequence alignment editor and analysis workbench. Bioinformatics.

[53] Lee S., Kim B.-D., Rose J.K.C. (2006). Identification of eukaryotic secreted and cell surface proteins using the yeast secretion trap screen. Nat. Protoc..

[54] Catlett N.L., Lee B., Yoder O.C., Turgeon B.G. (2003). Split-marker recombination for efficient targeted deletion of fungal genes. Fungal Genet. Rep..

[55] Kohn L.M., Stasovski E., Carbone J., Anderson J. (1991). Mycelial incompatibility and molecular markers identify genetic variability in field populations of *Sclerotinia sclerotiorum*. Phytopathology.

[56] Zhao H., Zhu Z., Xu Y., Wang H., Xie J., Cheng J., Jiang D., Fu Y. (2025). SsNEP2 plays a role in the interaction between *Sclerotinia sclerotiorum* and *Coniothyrium minitans*. J. Fungi.

