# Supplementary material for: The Function of Chitinases CmCH1 and CmCH10 in the Interaction of Coniothyrium minitans and Sclerotinia sclerotiorum
Source: Int J Mol Sci. 2025 Sep 6;26(17):8706. doi: 10.3390/ijms26178706 (PMC12428990; doi:10.3390/ijms26178706)
Supplement: Supplementary file 1 [file ijms-26-08706-s001.zip › ijms-3816844-supplementary.pdf]

Supplementary Materials

Supplementary Tables

Table S1. List of primers used in the study

Primer name	Primers sequence (5′ to 3′)	Description
5′AOX1	GACTGGTTCCAATTGACAAGC	Eukaryotic expression
3′AOX1	GCAAATGGCATTCTGACATCC	Eukaryotic expression
Bar-F	TCTGCACCATCGTCAACCACTA	Verifying *Bar*
Bar-R	TGAAGTCCAGCTGCCAGAAAC	Verifying *Bar*
BR-UF	AGAAGATGATATTGAAGGAGCACTTTT	*Bar* split segment
BR-UR	AGAAACCCACGTCATGCCAGTT	*Bar* split segment
CmActin-F	GATTGGTATGGGTCAGAA	*Actin* of *C. minitans*
CmActin-R	ATCTGGGTCATCTTCTCA	*Actin* of *C. minitans*
CmCH10-DDR	GTACACCTCGGGCTTAGTTTATGAA	*CmCH10* down down-stream
CmCH10-DF	gaagaaagcgaaagggctagcCGTTGCGTGTAAGAGATACCGA	*CmCH10* down-stream segment
CmCH10-DR	cgtcaatggctgcaggctagcATGTCGCTGGTCTGATTATTGTTAGA	*CmCH10* down-stream segment
CmCH10-SP-F	cggaattttaattaagaattcATGCGGTCGATAGCAATCCTC	Verifying signal peptide
CmCH10-SP-R	cactatagggagaacctcgagTGGAATAATAGCAGACGCCGA	Verifying signal peptide
CmCH10-UF	ttggtgactattgagcacgtgCTCGTGCAGAGGAGGCATCG	*CmCH10* up-stream segment
CmCH10-UR	ttaatcacgtatactcacgtgTGTCCCTGTATCGCAGTCTGTG	*CmCH10* up-stream segment
CmCH10-UUF	GCTGCGATGTAGAGCTTCTTTTTCA	Verifying *CmCH10* up up-stream
CmCH11-SP-F	cggaattttaattaagaattcATGCGTTCCTCACTCCTGTTGG	Verifying signal peptide
CmCH11-SP-R	cactatagggagaacctcgagGATTTTGAGAGCGAGGATCGAT	Verifying signal peptide
CmCH12-SP-F	cggaattttaattaagaattcATGCGCTCCCTTGTGGCT	Verifying signal peptide
CmCH12-SP-R	cactatagggagaacctcgagGAAAGTGACATTGGAGGCAAGC	Verifying signal peptide
CmCH1-DDR	GATGACTGCGGCAAGGACCA	Verifying *CmCH1* down down-stream
CmCH1-DF	tctagaggatccccgggtaccTTGGGAGGCTTGTGGTTGTC	*CmCH1* down-stream segment
CmCH1-DR	tacgaattcgagctcggtaccATGGCGACGGAGTTCAGGG	*CmCH1* down-stream segment
CmCH1-SP-F	cggaattttaattaagaattcATGATGCTCTTTGCTCTTGTATCAA	Verifying signal peptide
CmCH1-SP-R	cactatagggagaacctcgagAGCGAGCGCCTCGGAAAG	Verifying signal peptide
CmCH1-UF	acgacggccagtgccaagcttGGAGAAGCTGCTAAACTACCGC	*CmCH1* up-stream segment
CmCH1-UR	gacctgcaggcatgcaagcttGTGGGCGTTGTGAACTGGTG	CmCH1 up-stream segment
CmCH1-UUF	CTCGGATGATGCCTGATACGC	Verifying *CmCH1* up up-stream
CmCH4-SP-F	cggaattttaattaagaattcATGCCGGGTGCACGTGCC	Verifying signal peptide

Primer name	Primers sequence (5′ to 3′)	Description
CmCH4-SP-R	cactatagggagaacctcgagGCAGACGGCGGGTGAAGA	Verifying signal peptide
CmCH5-SP-F	cggaattttaattaagaattcATGGCTTTTCGGATGCTGG	Verifying signal peptide
CmCH5-SP-R	cactatagggagaacctcgagAGCCTGAGTTGTCTTCACACCG	Verifying signal peptide
CmCH6-SP-F	cggaattttaattaagaattcATGCGATCACCCATCACTCC	Verifying signal peptide
CmCH6-SP-R	cactatagggagaacctcgagTCCAAGTGCGGGCCAAAA	Verifying signal peptide
CmCH7-SP-F	cggaattttaattaagaattcATGTTCTCTCGATCTCTCGCCC	Verifying signal peptide
CmCH7-SP-R	cactatagggagaacctcgagTGCATCAAATGCTGAGACGG	Verifying signal peptide
CmCH8-SP-F	cggaattttaattaagaattcATGGGCATCTTTACATCCTTCTCG	Verifying signal peptide
CmCH8-SP-R	cactatagggagaacctcgagCCCTGCGTGAACAGAGGCT	Verifying signal peptide
CmCH9-SP-F	cggaattttaattaagaattcATGCATTTCACTTCGTTATCAGCT	Verifying signal peptide
CmCH9-SP-R	cactatagggagaacctcgagGAAACGCGCATGTACAGCTG	Verifying signal peptide
g-CmCH10-F	TTCCACAAGCCATACCCATC	Verifying *CmCH10*
g-CmCH10-R	GGGGTTTTGTTCGGAGTGG	Verifying *CmCH10*
g-CmCH1-F	CTTTGCCTCAGTCGCCTAAGAAC	Verifying *CmCH1*
g-CmCH1-R	GGTGAAGCGACAGTGAGAAG	Verifying *CmCH1*
HY-F	AGAAGATGATATTGAAGGAGCACTTTT	*HYG* split segment
HY-R	TCTGCTGCTCCATACAAGCCA	*HYG* split segment
OE-CmCH1-F	accttcaaagagctcactagtATGATGCTCTTTGCTCTTGTATCA A	Overexpression of *CmCH1*
OE-CmCH1-R	tgtctcaaccccgggggtaccGTTGTTGGGGAAGCCAGACC	Overexpression of *CmCH1*
pPIC9A-CH1-F	gctgaagcttacgtagaattcTCAATTCTCCCACTGTTGGGTC	Eukaryotic expression
pPIC9A-CH1-R	cgcggccgccctagggaattcATGATGATGATGATGATGGTTGT TG	Eukaryotic expression
qCH10-F	TCCTCACCCAGTCATTGCGGAACT	qRT-PCR
qCH10-R	GTGGACAGCCATAAGCAGGGTAA	qRT-PCR
qCH11-F	CGTCTCCGAGTCGTTTTGTCT	qRT-PCR
qCH11-R	CGCCGATGTCAGCGTGTATT	qRT-PCR
qCH12-F	TCAACCACGACCGCCACC	qRT-PCR
qCH12-R	TTATGGTGACTGCGGTGGG	qRT-PCR
qCH13-F	GCCGACCAAATCCGAAGAC	qRT-PCR
qCH13-R	AACCCGTCGCAGACTTAGATACT	qRT-PCR
qCH14-F	CTCCAAGGAAGCGGCGTG	qRT-PCR
qCH14-R	GCGATGAGGCGGGTTATG	qRT-PCR
qCH15-F	CTGTCGTCCTACTGGTATCTGCG	qRT-PCR
qCH15-R	CAGACCACACTGACCAGAACAAT	qRT-PCR
qCH1-F	GACGGTCTTGACATTGACTGGGA	qRT-PCR
qCH1-R	GGGTGAAGCGACAGTGAGAA	qRT-PCR
qCH2-F	TGCCTTCATCATACTCTACAACACA	qRT-PCR
qCH2-R	TTGCTCGTAAAGACTTCGTTGAGG	qRT-PCR
qCH3-F	CCCTCCACTTGTCAAAGACGT	qRT-PCR
qCH3-R	CTCTGAAGCCGTCCTCGTTACC	qRT-PCR
qCH4-F	GTGAGTATAGATGGGGCGGTTA	qRT-PCR
qCH4-R	AGGATTGCCAAAGGTTTTTGAT	qRT-PCR

Primer name	Primers sequence (5′ to 3′)	Description
qCH5-F	TTGCGAGACAAGTTCTAAGTTTCC	qRT-PCR
qCH5-R	CAGGAAGTGGTAGTCTGGGTGGT	qRT-PCR
qCH6-F	AAGGAGATGACTCAGACACGG	qRT-PCR
qCH6-R	ACAACGAGGAACAATAGGACG	qRT-PCR
qCH7-F	GAGGTTATCCCACCGACTACTA	qRT-PCR
qCH7-R	CTCGTAGTTGGCAGACAAGTATC	qRT-PCR
qCH8-F	AATTTTACAACAACGGATGTGGTG	qRT-PCR
qCH8-R	TCCATGAGCGTGCGAAGAC	qRT-PCR
qCH9-F	CAACACCGCTGCTTTCCA	qRT-PCR
qCH9-R	ATAGTGTCACCCCATCCGCC	qRT-PCR
RA-DF	GCACGCAACGCCTACGACTG	*Bar* split segment
RA-DR	TCATGTAATTAGTTATGTCACGCTT	*Bar* split segment
YG-F	GATCGTTATGTTTATCGGCACTTTG	*HYG* split segment
YG-R	AAGAAGGATTACCTCTAAACAAGTGTACC	*HYG* split segment
YZ-HYG-F	AGAAGATGATATTGAAGGAGCACT	Verifying *HYG*
YZ-HYG-R	AAGAAGGATTACCTCTAAACAAGTGT	Verifying *HYG*

Note: Lowercase letters in the table represent homologous arms in the corresponding carriers.

Table S2. Chitinase information of *C. minitans*

ID	Gene Length (bp)	CDs	Intron	Amino acid	Signal peptide	Molecular mass (kDa)	isoelectric point
CMZSB_00640 (CmCH1)	1384	1332	1	443	1-20	47.8	4.96
CMZSB_00643 (CmCH2)	2688	2688	-	895	-	94.5	4.32
CMZSB_01312 (CmCH3)	1202	1092	1	363	-	40.8	6.18
CMZSB_02023 (CmCH4)	1720	1596	-	531	1-22	58.3	5.49
CMZSB_02287 (CmCH5)	4931	4035	8	1344	1-25	140.5	4.81
CMZSB_02923 (CmCH6)	6881	3945	-	1314	1-22	142.0	4.90
CMZSB_04383 (CmCH7)	3188	3090	1	1029	1-17	106.0	9.13
CMZSB_05243 (CmCH8)	1378	1038	4	345	1-19	37.0	6.71
CMZSB_05256 (CmCH9)	1764	1542	4	513	1-18	54.8	4.32
CMZSB_06256 (CmCH10)	2229	1956	4	651	1-17	68.5	8.22
CMZSB_08378 (CmCH11)	2273	1185	3	394	-	43.5	5.93
CMZSB_08719 (CmCH12)	2204	1668	-	555	1-17	54.9	7.9
CMZSB_08720 (CmCH13)	5900	4686	3	1561	1-15	163.2	5.11
CMZSB_11210 (CmCH14)	1298	1239	1	412	-	44.8	5.28
CMZSB_11311 (CmCH15)	374	327	1	108	-	12.8	5.49

Supplementary Figures

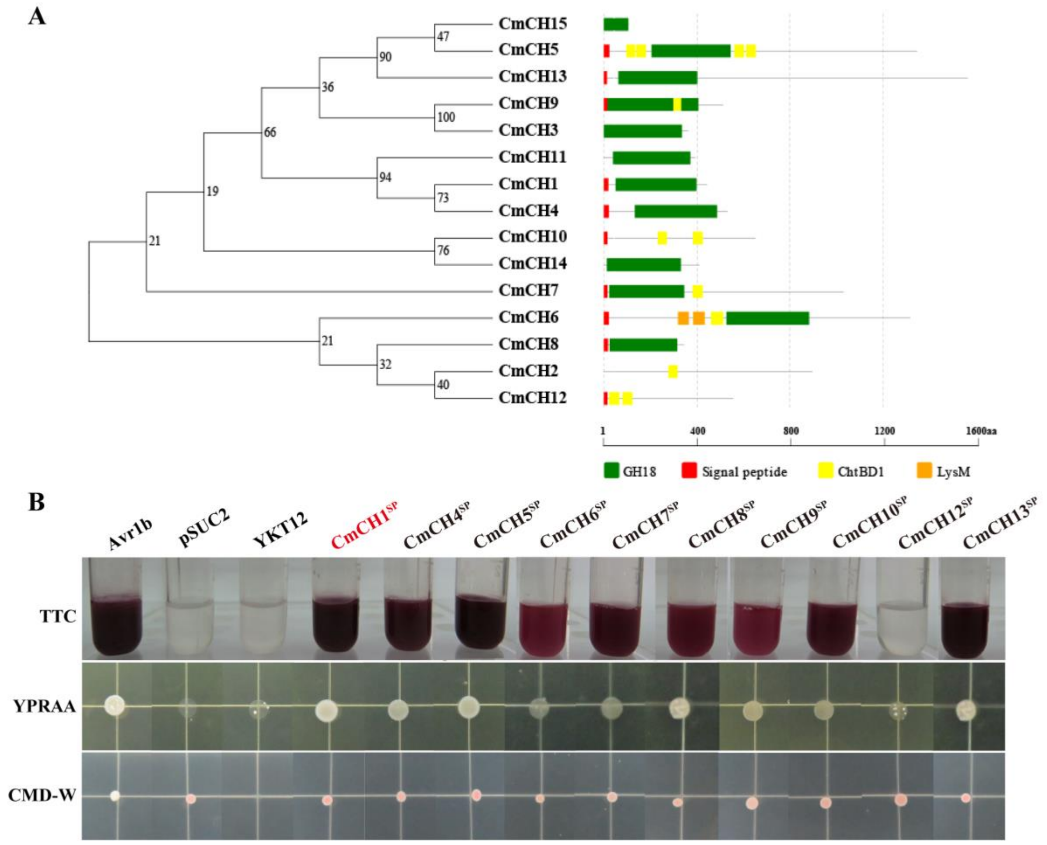

Figure S1. Phylogenetics and secretion activity of chitinase family-related proteins in *C. minitans.* (A) Phylogenetic tree and proteins structure of fifteen chitinases in *C. minitans*. Different colors were used to represent domain information, with green representing the catalytic region of GH18 family catalyzing chitin hydrolysis, red representing signal peptides, yellow representing the chtBD binding region, and orange representing the LysM domain. (B)TTC appears red or reddish-brown indicating that the signal peptide can perform its secretory function normally. Functional signal peptides enabled yeast growth on YPRAA and CMD-W. All yeast strains containing the pSUC2 vector can grow on CMD-W, and only secreting yeast strains can grow on YPRAA. The functional signal peptide of AVR1b served as a positive control, while strain YTK12 with the empty pSUC2 plasmid as a negative control.

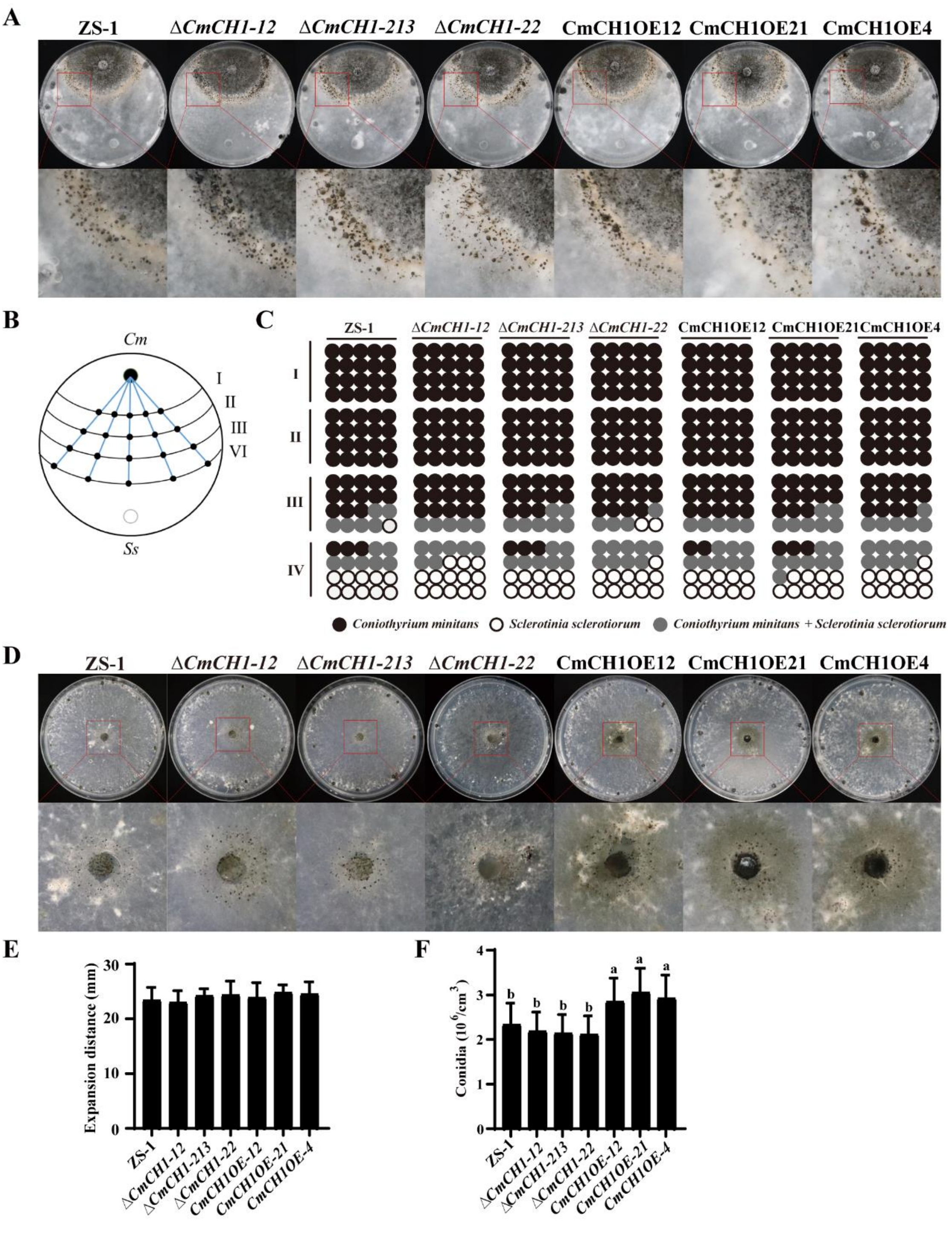

Figure S2. Mycelial parasitism of *CmCH1* mutants to *S. sclerotiorum*. (A) The colony morphology of *CmCH1* mutants co-cultured with *S. sclerotiorum* (20℃, 30 d). (B) Schematic depicting the sampling method for mycelial plugs from four consecutive zones (I–IV) in a dual culture of *C. minitans* and *S. sclerotiorum*. From the inoculation point of *C. minitans*, four 5-mm-wide zones were established at 10-mm intervals. Five plugs (5 mm in diameter) were collected from each zone, transferred to fresh PDA plates, and incubated for 7 days to determine fungal composition. (C) The results of punch sampling of *CmCH1* mutants cultured in each area. Each circle represents a colony developed from a mycelial agar disk sampled from zones I, II, III or IV between the inoculation sites of *C. minitans* and *S. sclerotiorum* in a dual-culture. The black circle presence of *C. minitans* colonies suggested that *S. sclerotiorum* was parasitized by

C. minitans, whereas the white circle appearance of *S. sclerotiorum* colonies indicated that *C. minitans* could not parasitize *S. sclerotiorum* in this region. The gray circle presence of of colonies of both *C. minitans* and *S. sclerotiorum* indicated that *C. minitans* was existed at this region but had not completely killed the *S. sclerotiorum*. (D) Parasitism of *CmCH1* mutants on colony of strain 1980 (20℃, 30 d). (E) The expansion distance of of *CmCH1* mutants on colony of strain 1980. (F) The conidial production of *CmCH1* mutants on colony of strain 1980. All values are represented as the average derived from multiple repetitions, with error bars representing ± SD of the average. Different lowercase letters (a, b) indicate significant differences between strains. One-way ANOVA, $P < 0.05$, n = 8.

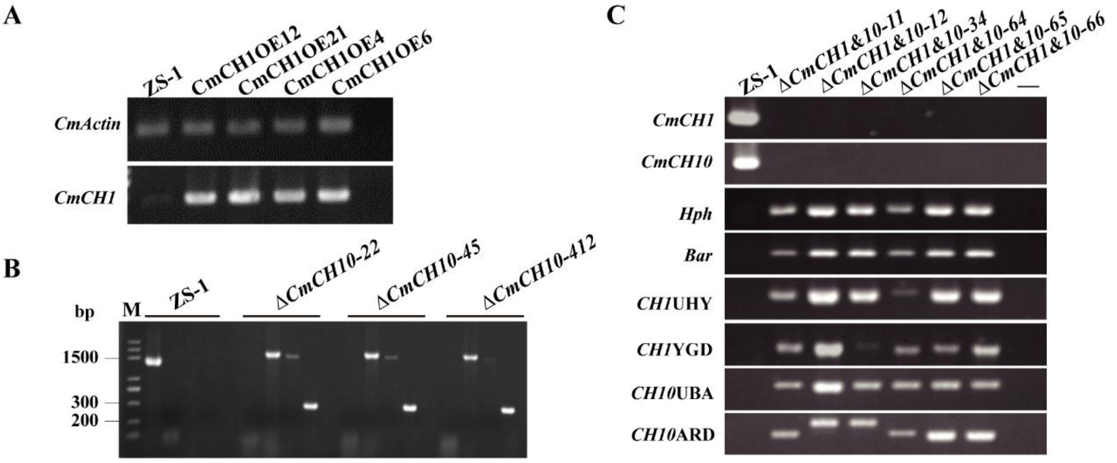

Figure S3. Validation of mutants. (A) RT-PCR Validation of *CmCH1* overexpression mutants. Using *Actin* as the internal reference gene to assess cDNA quality, the content of *CmCH1* in each strain was detected by PCR amplification with 28 cycles. The content of *CmCH1* in the overexpression mutants was significantly higher than that in ZS-1. (B) Validation of *CmCH10* knockout mutants. *CmCH10* could be amplified in strain ZS-1. In the knockout mutants, *CmCH10* could not be amplified, but the *Bar* and the upper and lower arms of *CmCH10* adjacent to the resistance gene could be amplified. (C) Validation of *CmCH1&10* mutants. *CmCH1* and *CmCH10* could be amplified in strain ZS-1, but could not be amplified in the double knockout mutants. Simultaneously, *Hph*, *Bar* and the original upper and lower arms connected to the resistance genes could be amplified in the mutants.

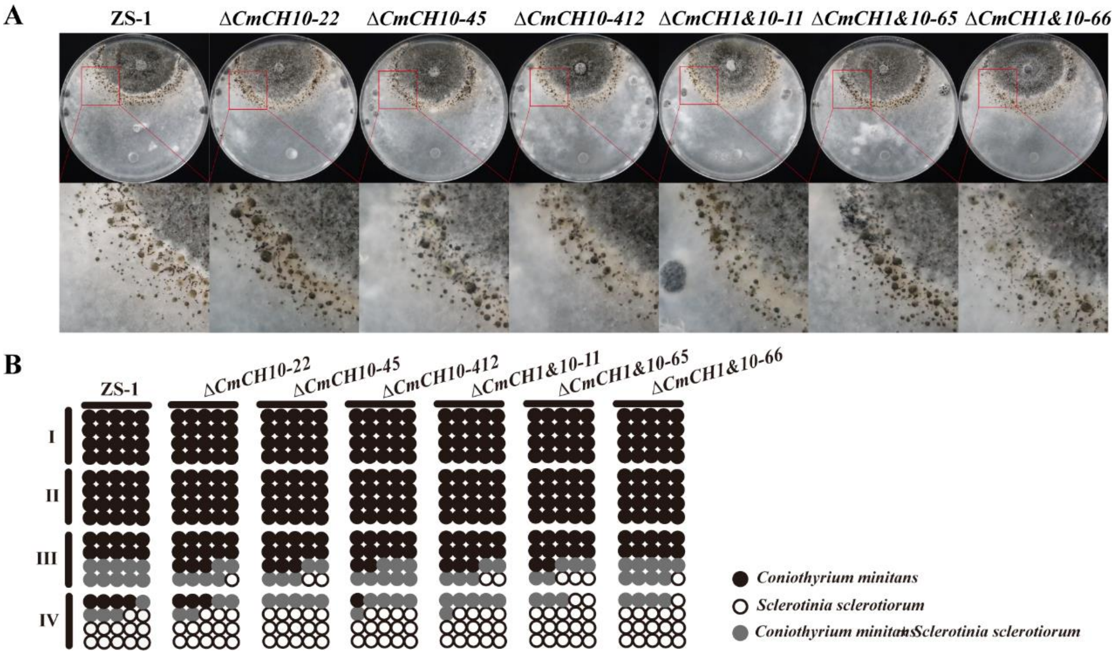

Figure S4. Parasitism of *CmCH1* and *CmCH1&10* mutants to mycelial of *S. sclerotiorum*. (A) The colony morphology of *CmCH10* and *CmCH1&10* mutants co-cultured with *S. sclerotiorum* on PDA (20°C, 30 d). (B) The results of punch sampling of *CmCH10* and *CmCH1&10* mutants cultured in each area.

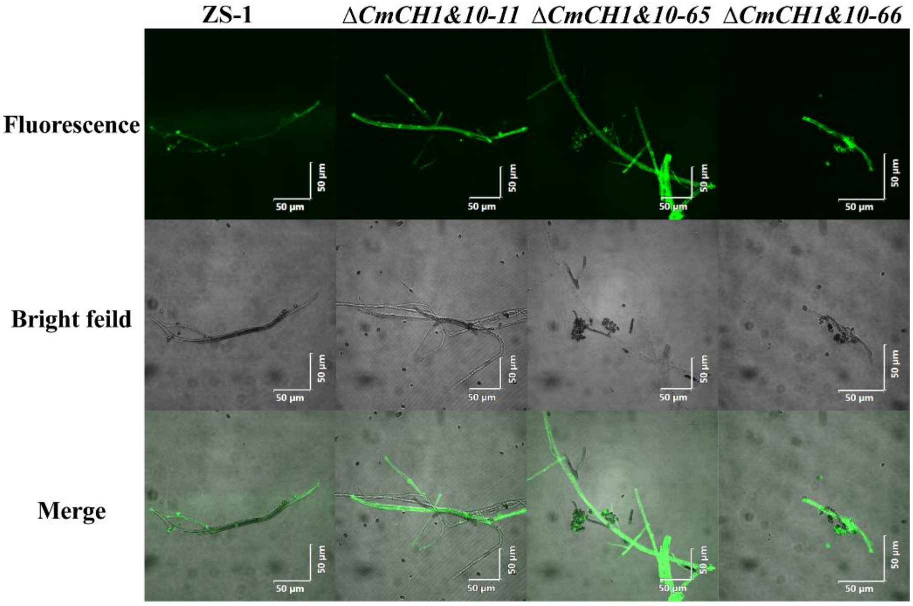

Figure S5. WGA488 staining of *CmCH1&10* double knockout mutants. Staining observation of chitin in the cell walls of *CmCH1&10* double knockout mutants and ZS-1 mycelium using 10 μg/mL WGA488. Bar = 50 μm.

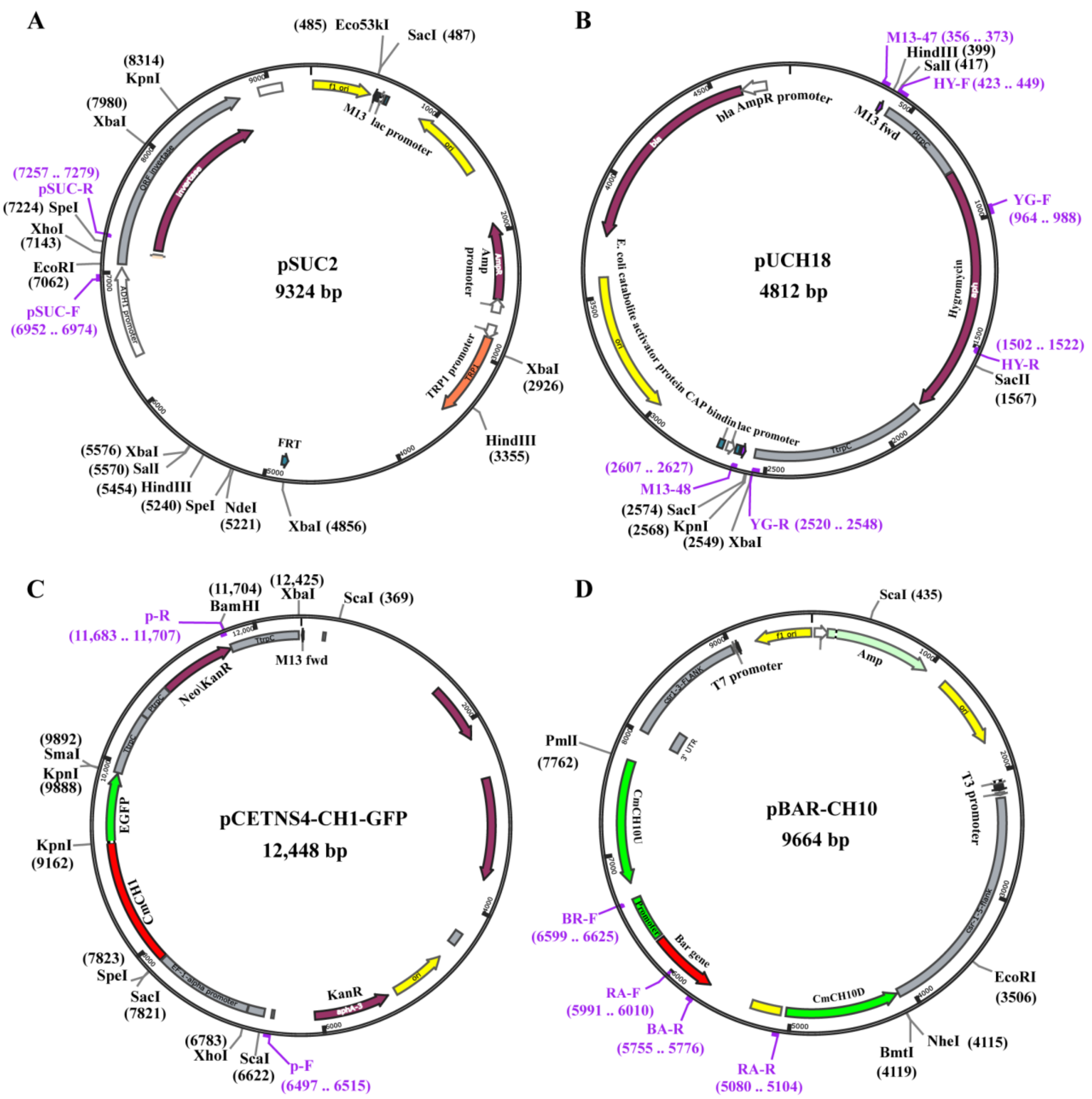

Figure S6. The vector maps involved in this article. (A) The map of the vector pSUC2 used for verifying the signal peptide. This vector has ampicillin resistance. (B) The map of the vector pUCH18 used for knocking out *CmCH1*. This vector has ampicillin resistance and contains a hygromycin resistance gene. (C) The map of the vector pCETNS4 used for overexpressing *CmCH1*. This vector has neomycin resistance, contains a kanamycin resistance gene and also contains a GFP gene. (D) The map of the vector pBAR used for knocking out *CmCH10*. This vector has ampicillin resistance and contains a herbicide resistance gene. In all the maps, the bright purple indicates the positions of primers, and the black indicates the relevant restriction enzyme cutting sites.
